# An Investigation into the Phytochemical Content and Antioxidant, Antidiabetic, and Wound-Healing Activities of *Curculigo latifolia* Found in Brunei Darussalam

**DOI:** 10.1155/2024/5656744

**Published:** 2024-08-03

**Authors:** Amanina Yusrina Taufik, Hartini Mohd Yasin, Norhayati Ahmad, Masayoshi Arai, Fairuzeta Ja'afar

**Affiliations:** ^1^ Chemical Sciences Faculty of Science Universiti Brunei Darussalam, Jalan Tungku Link, Gadong BE1410, Brunei Darussalam; ^2^ Osaka University ASEAN Campus Brunei Darussalam, No. 13, Kg Mabohai, Bandar Seri Begawan BA1111, Brunei Darussalam; ^3^ Environmental and Life Sciences Faculty of Science Universiti Brunei Darussalam, Jalan Tungku Link, Gadong BE1410, Brunei Darussalam; ^4^ Institute for Biodiversity and Environmental Research Universiti Brunei Darussalam, Jalan Tungku Link, Gadong BE1410, Brunei Darussalam; ^5^ Graduate School of Pharmaceutical Sciences Osaka University, 1–6 Yamadaoka, Suita, Osaka 565–0871, Japan

## Abstract

This present study aimed to investigate the phytochemical content and antioxidant and antidiabetic activities of *Curculigo latifolia* leaves (CL) and *C. latifolia* roots (CR) found in Brunei Darussalam. Phytochemical screening showed that CL and CR extracts contain saponins, tannins, glycosides, and terpenoids. CR showed higher total phenolic content (TPC), but lower total flavonoid content (TFC) when compared to CL. The high TPC in CR contributed to its potent radical scavenging activity (RSA) against 2,2-diphenyl-1-picrylhydrazyl (DPPH) radicals and strong ferric reducing antioxidant power (FRAP). Additionally, CR exerted significant inhibition of ∝-glucosidase and ∝-amylase, suggesting a potential link between the chemical compounds and its antioxidant and antidiabetic effects. In the animal study of antihyperglycemic activity, treatment with 250 mg/kg body weight (b.w.) of the CL extract normalised the blood glucose levels and improved body weight gain of alloxan-induced diabetic rats within 14 weeks. Furthermore, our investigation into the wound-healing effects of young *C. latifolia* leaves (YCL) and matured *C. latifolia* leaves (MCL) showed a significant reduction in wound size on Day 3, 5, and 7 of the experimental study, indicating its wound-healing potential. Based on our findings, *C. latifolia* can be consumed as part of a balanced diet due to its antioxidant and antidiabetic properties.

## 1. Introduction

Diabetes is a serious chronic medical condition that occurs when a body experiences unregulated blood glucose levels [1]. This disease is usually associated with deficiency in insulin secretion, insulin action, or both [1]. There are two main types of diabetes, which are type 1 diabetes and type 2 diabetes. Patients with type 1 diabetes usually suffer from absolute deficiency of insulin that results from an autoimmune destruction of pancreatic *β*-cells [2]. Meanwhile, type 2 diabetes occurs when there is insulin resistance or when the pancreas does not produce enough insulin [2]. The prevalence of diabetes is increasing over the years, and it poses as a significant risk with various complications such as cardiovascular disease, kidney failure, nerve damage, and vision problems [3]. Chronic wound (foot ulcers) due to impaired healing is another microvascular complication in diabetes, resulting from physiological processes encompassing vascular, neuropathic, immune, and biochemical elements [4]. Additionally, poor circulation of blood, uncontrolled hyperglycemia, and prolonged inflammation cause a significant delay in the wound-healing process, subsequently increasing the risk of infection, tissue necrosis, gangrene, and nontraumatic amputation [5]. The management of diabetic foot ulcers poses a medical and clinical challenge, and therefore, advancements in wound care technology and diabetes treatment are essential for an enhanced quality of life for affected individuals.

Medicinal plants have gained recognition for their therapeutic properties such as antidiabetic, anti-inflammatory, wound-healing, and antimicrobial activities [6]. Brunei Darussalam has rich biodiversity and a wide range of flora and fauna, many of which have been often utilised in traditional medicine [7]. Despite their longstanding traditional use, scientific evidence supporting their therapeutic efficacies is still limited. One of the local plants that has been recognised for its healing attributes is *Curculigo latifolia*, or locally known as lemba, which belongs to the Hypoxidaceae family and genus *Curculigo* [8]. The plant is commonly found in the Borneo Island and Southeast Asia regions and is traditionally used to treat headaches, mouth thrush, and diarrhoeas [9]. A previous study found the root extract to contain various bioactive compounds, such as phloridzin, pomiferin, and cinnamic acid, which exhibited antidiabetic activities through reducing blood glucose levels and increasing insulin secretion [10]. There have also been reports that revealed that the fruit, root, and leaf extracts of *C. latifolia* were able to stimulate glucose uptake activity in 3T3-L1 adipocytes and L6 myotube cell lines [10]. Additionally, the extracts were shown to have an insulin-mimicking action that could activate insulin signaling cascade and enhance basal glucose uptake [10]. *C. latifolia* shows potential in supporting health improvements that could be useful in the treatment of various diseases, such as diabetes, inflammation, and cancer. Furthermore, the plant could serve as a valuable source of dietary supplementation that can contribute to overall health and vitality.

In this study, we aim to investigate the phytochemical contents and antioxidant and antidiabetic activities by chemical assays of *C. latifolia* leaves and *C. latifolia* roots. Additionally, the study also investigated the wound-healing activity of young and matured *C. latifolia* leaves on animal models at high and low doses.

## 2. Materials and Methods

**Figure 1:**
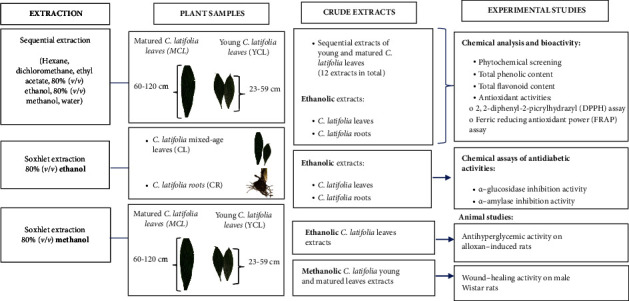
Methodology flowchart.

In this paper, we evaluated the phytochemical contents and antioxidant activities of young (YCL) and matured (MCL) leaves of *C. latifolia* extracted in different solvents (*n*-hexane, dichloromethane, ethyl acetate, 80% (*v/v*) ethanol, 80% (*v/v*) methanol, and water), as well as ethanolic *C. latifolia* mixed-aged leaf (CL) and *C. latifolia* root (CR) extracts. Subsequently, ethanolic CL extract was further assessed for its antidiabetic activity, while methanolic YCL and MCL extracts were investigated for their wound-healing activity on male Wistar rats. The experimental design of this study is further illustrated in Figure 1.

### 2.1. Plant Collection and Sample Preparations

**Figure 2:**
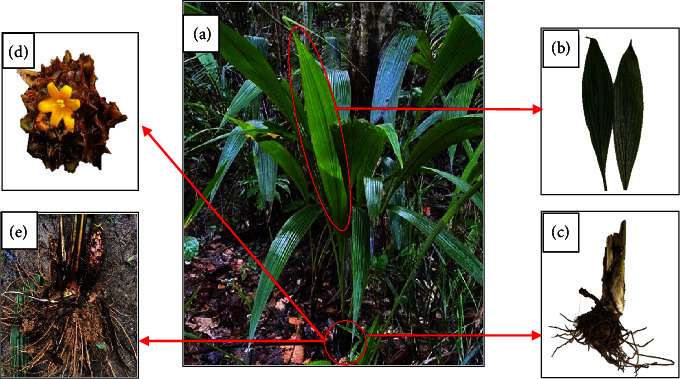
(a) *Curculigo latifolia* group plantation in Kuala Belait, Brunei Darussalam. (b) Leaves, (c) roots, (d) flower, and (e) underground part of *Curculigo latifolia* (photos taken by Amanina Yusrina Taufik, date: 19/12/2021).

*C. latifolia* leaves (matured and young) and roots were collected from Kampong Tenajor, Labi in Daerah Belait, Brunei Darussalam (4°27′34.1″N, 114°28′09.2″E). Species were identified and confirmed by UBD Botanical Research Centre (BRC) botanists, and a voucher specimen number B 038 880 was archived in Brunei Herbarium Sungai Liang.  Figure 2 shows the *C. latifolia* group plantation found in Kuala Belait in Brunei Darussalam.

**Figure 3:**
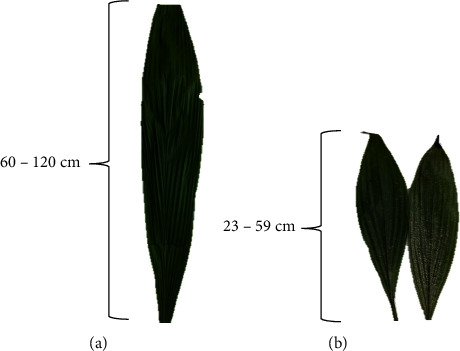
(a) Matured and (b) young leaves of *Curculigo latifolia*.

The collected samples were washed with tap water to remove any remaining dirt and subsequently air-dried at room temperature for 4 weeks until a constant mass was reached. For the *C. latifolia* leaves, they were arranged according to their respective sizes, where large leaves between 60 and 120 cm were categorised as matured leaves, and smaller leaves in the range of 23–59 cm were labelled as young leaves (see Figure 3). Subsequently, the dried samples were pulverised using a kitchen blender (Zojirushi, BM–RSQ08) and sieved using 365-micron siever. Powdered samples were stored in desiccators at room temperature until further use.

### 2.2. Preparation of Crude Extracts by Soxhlet and Sequential Extractions

Sequential extraction using *n*-hexane, dichloromethane, ethyl acetate, 80% (*v/v*) ethanol, 80% (*v/v*) methanol, and water was conducted on individual *C. latifolia* young and matured leaves. However, in the next part of the study, *C. latifolia* young and matured leaves were combined due to their limited amount and to simplify the collection process.

#### 2.2.1. Sequential Extraction

Different solvents of increasing polarity were used in sequential extraction. The powdered samples of young and matured leaves of *C. latifolia* (50 g) were initially soaked in *n*-hexane (1 L) and shaken for 3 hours (SF1 Flask Shaker, Stuart Equipment, Staffordshire, United Kingdom), followed by 3 hours of sonication (Elmasonic E, E 180 H, Germany) at a constant temperature of 35°C, and subsequently, filtered via vacuum filtration. With the residue obtained, the same procedure was repeated, following a predefined solvent sequence in the order of dichloromethane, ethyl acetate, 80% (*v/v*) ethanol, 80% (*v/v*) methanol, and water. The use of different solvents was aimed to extract compounds with a range of polarities, from low to high. All extractions were conducted under a constant temperature of 35°C, except for the solvent dichloromethane, which was maintained at 25°C due to its lower boiling point. Consequently, from each filtrate, the solvent was evaporated using rotary evaporator and dried in an oven at 35°C until a constant mass was obtained. The dried crude extracts were stored in an airtight container and kept in desiccators at room temperature until further use.

#### 2.2.2. Soxhlet Extraction

Powdered *C. latifolia* leaves (mixed-age) (50 g) and *C. latifolia* roots (50 g) were extracted in 80% (*v/v*) ethanol (1 L) in a Soxhlet extractor. Moreover, an additional investigation involved extracting individual *C. latifolia* young and matured leaves (50 g) in 80% (*v/v*) methanol (1 L). The extraction cycle was repeated for 2-3 days or until the solvent in the Soxhlet extractor turned colourless. Subsequently, the solvent was evaporated using a rotary evaporator and dried in the oven at 35°C until a constant mass was reached. The dried crude extracts were kept in airtight sample vials and placed in a desiccator at room temperature prior to use.

### 2.3. Phytochemical Contents by Chemical Assays

Our study involved qualitative screening for phytochemicals and quantitative analyses of total phenolic and flavonoid contents on ethanolic extracts of *C. latifolia* leaves (mixed-age), *C. latifolia* roots, and all of the sequential extracts of young and matured *C. latifolia* leaves.

#### 2.3.1. Qualitative Phytochemical Screening

The phytochemical screening of the different extracts was carried out to confirm the presence of phytochemicals such as alkaloids, saponins, tannins, terpenoids, steroids, and glycosides. The results are further presented as (+) and (−) denoting the presence and absence of the phytochemicals, respectively.

*(1) Test for Alkaloids*. The presence of alkaloids was tested according to the method described by Tadesse et al. [11] with minor modifications. Each extract (20 mg) was dissolved in 1% (*v/v*) hydrochloric acid (8 mL) and filtered. The filtrate attained was then mixed with 1 mL of potassium bismuth iodide solution (Dragendorff's reagent), where formation of orange red precipitate shows the presence of alkaloids.

*(2) Test for Saponins*. The extracts were screened for the presence of saponins using the methods outlined by Auwal et al. [12]. Each extract (50 mg) was mixed with 1 mL of distilled water in a test tube and the mixture was shaken vigorously. In the presence of saponins, formation of stable frothing would be observed.

*(3) Test for Tannins*. The screen test for tannins was investigated with the procedures detailed by Auwal et al. [12]. Each extract (50 mg) was dissolved in distilled water (1 mL) before drops of 5% (*w/v*) ferric chloride were added to the mixture. The formation of black or blue-green precipitate shows that tannins are present.

*(4) Test for Terpenoids*. The extracts were each tested for the presence of terpenoids according to the method by Tadesse et al. [11]. The test, namely, Salkowski test, was conducted by mixing the extract (50 mg) together with 1 mL of chloroform, followed by 1 mL of concentrated sulfuric acid, which was poured slowly along the sides of the test tube.

*(5) Test for Glycosides*. For identification of glycoside, the Keller–Killiani test was performed using the methods described by Gul et al. [13] with slight modifications. Each extract (50 g) was mixed with 5 mL of distilled water, followed by an addition of 2 mL of glacial acetic acid, together with 2-3 drops of ferric chloride and 1 mL of concentrated sulfuric acid along the sides of the test tube. A positive result for the presence of glycoside would give a formation of brown or velvet ring.

#### 2.3.2. Total Phenolic Content

The Folin–Ciocalteu colorimetric method described by Aryal et al. [14] was conducted with some modifications, to determine the total phenolic content (TPC). An aliquot of 0.3 mL of extract was mixed with Folin–Ciocalteu phenol's reagent (2.5 mL) and after 5 minutes, 6% (*w/v*) sodium carbonate (2.5 mL) was added. The mixture was incubated for 30 minutes and subsequently measured for its absorbance at wavelength 725 nm using a single beam UV-visible spectrometer (GENESYS™ 20S, Thermo Fisher Scientific Inc., USA). The standard calibration curve for gallic acid (0–100 mg/L) was prepared in the same manner and the TPC of extracts was attained using equation (1), where the results were expressed as gallic acid equivalence (GAE) in mg/g of extract.

(1) $$\begin{array}{c} \text{TPC in}\frac{\text{mg GAE}}{g\text{ of extract}}=\frac{\left[\text{Gallic acid}\right]\text{ in mg}/L\times \text{volume of extract}\left(L\right)}{\text{mass of extract }\left(g\right)}. \end{array}$$

#### 2.3.3. Total Flavonoid Content

Total flavonoid content (TFC) was determined using the aluminium colorimetric method described by Salim et al. [15] with some modifications. The extract solution (0.5 mL) was mixed with 10% (*w/v*) aluminium chloride (0.1 mL), 1 M potassium acetate (0.1 mL), absolute ethanol (1.5 mL), and distilled water (2.8 mL). The mixture was incubated for 30 minutes, and the absorbance was measured at wavelength 415 nm using a single beam UV-visible spectrometer (GENESYS™ 20S, Thermo Fisher Scientific Inc., USA). Subsequently, the TFC of extracts was obtained from the calibration curve of quercetin (1–100 mg/L), using equation (2), and the results were expressed as quercetin equivalence (QE) in mg/g of extract.

(2) $$\begin{array}{c} \text{TFC in }\frac{\text{mg QE}}{g\text{ of extract}}=\frac{\left[\text{Quercetin}\right]\text{ in mg}/L\times \text{volume of extract}\left(L\right)}{\text{mass of extract used }\left(g\right)}. \end{array}$$

### 2.4. Antioxidant Activities

The antioxidant activity of *C. latifolia* leaves (mixed-age), *C. latifolia* roots, and all the sequential extracts of young and matured *C. latifolia* leaves was investigated using 2,2-diphenyl-2-picrylhydrazyl (DPPH) and ferric reducing antioxidant power assays.

#### 2.4.1. 2,2-Diphenyl-2-picrylhydrazyl (DPPH) Assay

The DPPH radical scavenging activity was evaluated according to the methods by Adli et al. [16] with some modifications. Briefly, a stock solution of 50 mg/L DPPH was made fresh with absolute methanol prior to analysis. A standard gallic acid was prepared in concentrations between 0.5 mg/L to 10 mg/L, whereas the extract solutions were made into several concentrations between 10 and 100 mg/L. Subsequently, the DPPH stock solution (200 *µ*L) was added into each of the standard solutions and extract samples (25 *µ*L) and further kept in the dark for 30 minutes. The absorbance was then taken using a microplate reader (BIOBASE, BK–EL10C, China) at 517 nm against methanol as the negative blank. The percentage DPPH radical inhibition was calculated using the following equation.

(3) $$\begin{array}{c} \text{Percentage inhibition of DPPH radical }\left(\%\right)=\frac{\left(A_{ο}-A_{s}\right)}{A_{ο}}\times 100. \end{array}$$

The IC_50_ was obtained from the calibration curve of percentage inhibition against the concentrations of each extract or standard. Subsequently, the DPPH radical scavenging activity (RSA) was determined based on equation (4) and the results were expressed in mg gallic acid equivalent (GAE)/g extract.

(4) $$\begin{array}{c} \text{DPPH RSA in }\frac{\text{mg GAE}}{g\text{ of extract}}=\frac{{\text{IC}}_{50}\left(\text{Gallic acid}\right)\times 1000}{{\text{IC}}_{50}\left(\text{sample extract}\right)}. \end{array}$$

#### 2.4.2. Ferric Reducing Antioxidant Power (FRAP) Assay

For ferric ion reducing antioxidant power assay, the method used was by Pratami et al. [17] with modifications. Fresh FRAP reagent was prepared by mixing 300 mM acetate buffer (pH 3.6), acidified 2,4,6-tris(2-pyridyl)-s-triazine (TPTZ) solution (10 mM), and FeCl_3_·6 H_2_O (20 mM) at a ratio of 10 : 1:1 and subsequently warmed at 37°C. The sample extracts were prepared at 1000 mg/L, while the standard, Trolox, was diluted into a range of concentrations between 50 and 250 mg/L. An aliquot of 50 *µ*L of each Trolox concentration and sample extract was mixed with 950 *µ*L of the working FRAP reagent on a 96-well plate and the mixtures were incubated in darkness for 30 minutes at room temperature. Subsequently, the absorbance was measured at 593 nm using a microplate reader (BIOBASE, BK–EL10C, China) and a calibration curve of absorbance against Trolox concentration was plotted. The ferric reducing antioxidant power of the extracts was calculated using equation (5) and expressed in Trolox equivalence (TE) mg/g of extract.

(5) $$\begin{array}{c} \text{FRAP in}\frac{\text{mg TE}}{g\text{ of extract}}=\frac{\left[\text{Trolox}\right]\times \text{volume of extract}\left(L\right)}{\text{mass of extract used}\left(g\right)}. \end{array}$$

### 2.5. Antidiabetic Activity by Chemical Assays

The antidiabetic potential of ethanolic *C. latifolia* leaf (mixed-age) and *C. latifolia* root extracts was assessed through assays, including *α*-glucosidase and *α*-amylase inhibition activities.

#### 2.5.1. *α*-Glucosidase Inhibition Activity

The individual extracts were analysed for their *α*-glucosidase inhibition activity through the methods described by Nor et al. [18]. Sample extracts and positive control, acarbose (200 mg/L), were dissolved in 10% (*v/v*) dimethyl sulfoxide. Aliquots of 30 *µ*L *α*-glucosidase solution (1 U/mL) were added into the wells of a 96-well plate containing 50 *µ*L of different concentrations of samples and positive control. The mixtures were incubated in the dark at 37°C for 5 minutes and subsequently, 20 *µ*L of 0.02 M p-nitrophenyl-*α-D*-glucopyranoside (pNPG) substrate was added to each well and incubated for another 15 minutes in the same conditions. Following that, 50 *µ*L sodium carbonate (0.2 M) was added to stop the reaction and the absorbance was taken at wavelength 405 nm using 96-well microplate reader (BIOBASE, BK–EL10C, China). The percentage inhibition was obtained using equation (6), where *A*_*o*_ indicates the absorbance of control and *A*_*s*_ is the absorbance of samples.

(6) $$\begin{array}{c} \text{Percentage inhibition of }\alpha -\text{glucosidase }\left(\%\right)=\frac{\left(A_{ο}-A_{s}\right)}{A_{ο}}\times 100. \end{array}$$

The experiment was conducted in triplicate and the data obtained are expressed in mean ± standard deviation.

#### 2.5.2. *α*-Amylase Enzyme Activity

Amylase activity of the individual extracts was determined using a colorimetric method based on a commercialised amylase assay kit (catalog no. MAK009, Sigma-Aldrich, Merck, USA). Sample extracts and positive control, acarbose (200 mg/L), were diluted in Amylase Assay Buffer from the kit, and nitrophenol standards of varying concentrations of 0 (blank), 4, 8, 12, 16, and 20 nmol/well standards were prepared to obtain a linear range of calibration curve. Prior to assay reaction, Master Reaction Mix was made ready by mixing an equal ratio of Amylase Assay Buffer (50 *µ*L) and Amylase Substrate Mix (50 *µ*L) and then added into individual wells of 96-well plate containing 50 *µ*L of the extracts, standards, and positive control. The initial absorbance at 405 nm was measured [(A_405_)_initial_] using a 96-well microplate reader (BIOBASE, BK–EL10C, China), and the absorbance was recorded every 5 minutes for 35 minutes. The sample with the highest change in absorbance (calculated using equation (7)) was then used to determine the amylase activity (using equation (8)).

(7) $$\begin{array}{c} {∆A}_{405\text{nm}}={\left(A_{405}\right)}_{\text{final}}-{\left(A_{405}\right)}_{\text{initial}}, \end{array}$$

(8) $$\begin{array}{c} \text{Amylase activity }\left(\%\right)=\frac{n\left[\text{Nitrophenol}\right]\left(\text{nmol}\right)\times \text{Sample dilution factor}}{\text{Reaction time}\left(\min\right)\times \text{Volume}\left(\text{mL}\right)}\times 100. \end{array}$$

The number of moles of nitrophenol in the sample was evaluated from the standard calibration curve of nitrophenol. The amylase activity of the extracts and positive control was expressed as milliunit (mU), which is equivalent to nmol/min/mL. One unit of amylase refers to the amount of amylase that links ethylidene-pNP-G7 to produce 1.0 *μ*mol of p-nitrophenol per minute.

### 2.6. Animal Studies

Ethanolic extract of *C. latifolia* mixed-age leaves was investigated for its antihyperglycemic effects on alloxan-induced diabetic rats. Additionally, methanolic extracts of young and matured *C. latifolia* leaves were evaluated for their wound-healing potential on male Wistar rats.

#### 2.6.1. Antihyperglycemic Activity on Animal Model

Antihyperglycemic study was conducted based on the protocol described by Hazirah Matusin et al. [19]. In this preliminary study, *C. latifolia* leaf extract was chosen to evaluate its capacity to lower down blood glucose level (BGL) and changes in body weight percentage of alloxan-induced diabetic rats.

*(1) Experimental Animals*. Male Wistar rats weighing 160–200 g and aged between 8 and 10 weeks old from the animal house of Universiti Brunei Darussalam were used. The animals were housed in polypropylene cages that were cleaned regularly and were provided *ad libitum* access to water and standard pellet rat diet (Altronim, Germany). The procedures in this antihyperglycemic experimental study were approved by the University Research Ethics Committee (Ref File: UBD/FOS/E2. (j)). Upon completion of experimental procedures, animals were euthanised using carbon dioxide asphyxiation. The carbon dioxide was gradually filled into the euthanasia chamber at a flow rate between 30 and 70% of the chamber volume per minute or 400–1000 ppm, depending on the size of the rats. Animals were kept in the chamber for a few minutes until death was confirmed by checking their respiratory arrest or observing their fixed and dilated pupils.

*(2) Administration of Alloxan*. Animals were abstained from food for 16 hours and were only provided with water before administration of alloxan. 0.9% (*w/v*) saline was used to dissolve the alloxan monohydrate (Sigma-Aldrich, United Kingdom) and a single dose of 120 mg/kg body weight (b.w.) was administered through the intraperitoneal cavity part of the animal to instigate diabetes. Their blood glucose levels were observed 72 hours after administration of alloxan and animals with readings above 15.0 mmol/L were deemed to be diabetic and were used in the antihyperglycemic study.

*(3) Experimental Groups of Antihyperglycemic Study*. This research project is composed of three main groups, where each group consists of six animals: normal untreated control (*n* = 6), diabetic control group (*n* = 6), and treated with *C. latifolia* leaf extract group (*n* = 6). Normal untreated control consists of healthy normal male Wistar rats, while diabetic control involves animals induced with 120 mg/kg b.w. of alloxan and not treated with sample extract. Animals that showed high readings of blood glucose 72 hours after alloxan administration were chosen for this study and only received normal diet throughout the experiment. For the treatment group, oral gavage administration of *C. latifolia* leaf extract at 250 mg/kg b.w. was carried out on the animals twice a week over a period of 14 weeks. The dosage of extract was determined based on the method by Hazirah Matusin et al. [19]. Subsequently, body weight and blood glucose reading of each rat were recorded on a weekly basis. An acute toxicology observation on alloxan-induced animals treated with 250 mg/kg b.w. *C. latifolia* leaf extract demonstrated no signs of morbidity or mortality throughout the experiment, which suggested the dosage was safe to use.

*(4) Blood Glucose Level and Body Weight Percentage*. The animals in this study were regularly measured for their blood glucose level in mmol/L using blood glucose test strips and a glucometer (FreeStyle Optium, Abbot Diabetes Care Ltd.). Measurement of blood glucose level was obtained by drawing blood from the tail tips using a single touch strip. Nonfasting animals of normal control, diabetic control, and treatment groups were regularly recorded for their blood glucose levels for 14 weeks, for which readings above 11.1 mmol/L were considered diabetic. Simultaneously, body weights of the animals were also taken once a week using a digital weighing machine (BX22KH Model, Shimadzu Corporation) and the data obtained were expressed in body weight percentage (%).

*(5) Intraperitoneal Glucose Tolerance Testing (IPGTT)*. An IPGTT test was conducted on all the animals in normal control, diabetic control, and treatment groups after the final experimental period had ended. The animals were required to fast for 16–18 hours and subsequently were measured for their blood glucose reading; this time point was defined as 0 min. Each animal in every group was injected with a single dose of 2 g/kg b.w. D-glucose and their blood glucose levels were monitored at a time interval of 15, 30, 60, 90, and 120 minutes. Blood glucose readings were expressed as mmol/L.

#### 2.6.2. Wound-Healing Activity on Animal Model

Wound-healing activity was conducted based on the methods described by Shafie et al. [20]. Methanolic young and matured leaves of *C. latifolia* leaves were investigated for their wound-healing effects on male Wistar rats. This preliminary study measured the percent wound contraction of the animals after topical treatment of the extracts on alternate days for 14 days.

*(1) Experimental Animals*. In this research project, adult male Wistar rats of 8–10 weeks old, weighing about 200–400 g, from the animal house of Universiti Brunei Darussalam were used. The rats were individually placed in clean polypropylene cages and provided ad libitum access to food and water. Experimental procedures were approved by the University Research Ethics Committee (Ref file: UBD/FOS/N(1)). After the experiments were completed, animals were euthanised using carbon dioxide asphyxiation. The carbon dioxide flow rate was between 30 and 70% of the euthanasia chamber volume per minute or between 400 and 1000 ppm, depending on the size of the rats. Animals were maintained in the chamber for several minutes until death was confirmed by observing their fixed and dilated pupils and respiratory arrest.

**Figure 4:**
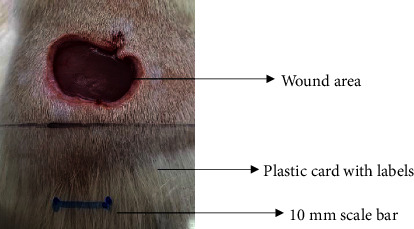
Layout of measuring the wound contraction of male Wistar rat.

*(2) Wound Excision*. Prior to excision procedure, the rats were individually anaesthetised with diethyl ether by inhalation anaesthesia. A few drops of diethyl ether were applied onto a cotton wool and then placed at the bottom of a transparent acrylic jar. Subsequently, the animals were exposed to the diethyl ether without direct contact between the cotton wool and the animal. The level of anaesthesia by exposure of diethyl ether was assessed by observing the animals' ability to make voluntary movements, such as extending their legs in response to stimuli. After ensuring deep anaesthesia of the animals, wound excision was performed. The dorsal thoracic region was shaved using an electric shaver (Surker, HD 8202, UK) and then cleaned with absolute ethanol before excising the skin (10 mm × 10 mm) using forceps and pointed scissors (see Figure 4). The wound area was further treated with saline, and the rats were then moved to a well-ventilated area to recover from the anaesthesia. Topical application of prepared ointments on wound area was done once in every 2 days intervals for a period of 14 days for each treatment group.

*(3) Wound Treatment and Wound Area Evaluation*. The rats were randomly distributed into 6 groups (*n* = 4 rats each): (1) Control Group 1 (untreated), (2) Control Group 2 (treated with 100% (*w/w*) Vaseline), (3) Group 3 (treated with high dose 50% (*w/w*) methanolic matured leaf (Me-MCL) extract in Vaseline), (4) Group 4 (treated with high dose 50% (*w/w*) methanolic young leaf (Me-YCL) extract in Vaseline), (5) Group 5 (treated with low dose 10% (*w/w*) Me-MCL extract in Vaseline), and (6) Group 6 (treated with low dose 10% (*w/w*) Me-YCL extract in Vaseline).

The ointment was regularly applied once in every 2 days intervals for 14 days and the wound size was observed on Day 1, 3, 5, 7, 10, 12, and 14. Furthermore, the wound area for each day was measured using ImageJ software (National Institute Health, USA) and the wound contraction was determined using the following equation.

(9) $$\begin{array}{c} \text{Wound contraction}\left(\%\right)=\frac{\text{Wound area on day }1\left({\text{mm}}^{2}\right)-\text{Wound area on day}\left(n\right)\left({\text{mm}}^{2}\right)}{\text{Wound area on day }1\;\left({\text{mm}}^{2}\right)}\times 100. \end{array}$$

### 2.7. Statistical Analysis

The data obtained were represented as mean ± standard error of mean (S.E.M.), and the statistical significance between the groups was determined using one-way analysis of variance (ANOVA) with post hoc Tukey HSD test. Results with *p* < 0.05 and *p* < 0.01 were considered as significant. Moreover, Pearson's correlation analysis was carried out to determine the linear correlations between TPC and TFC with DPPH antioxidant activity and FRAP activity. Results with *R* value >0.5 indicate a positive correlation.

## 3. Results

### 3.1. Extraction Yield of Crude Extracts

**Figure 5:**
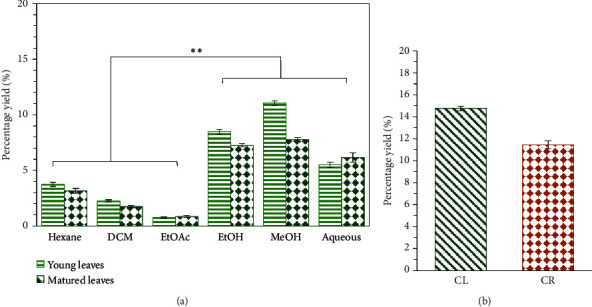
Average extraction yield of (a) sequential extracts and (b) ethanolic *C. latifolia* leaf (CL) and *C. latifolia* root (CR) extracts. Data are expressed as mean ± standard deviation (*n* = 3); ^*∗∗*^*p* < 0.01 indicates the significant difference between solvents of low polarity and high polarity.

Extraction yield of sequential and Soxhlet extracts of *C. latifolia* is presented in Figure 5. The results are presented as mean ± standard deviation of repeats (*n* = 3), in units of grams per 100 grams of original sample (g/100 g).

*C. latifolia* young leaves (YCL) and matured leaves (MCL) were extracted using various solvents starting from nonpolar to polar solvents (*n*-hexane, dichloromethane (DCM), ethyl acetate (EtOAc), 80% (*v/v*) ethanol (EtOH), 80% (*v/v*) methanol (MeOH), and water). Our results in Figure 5(a) demonstrated high polar solvents to be more effective in the extraction of YCL and MCL, with 80% (*v/v*) methanol having the highest yield (YCL, 11.03 ± 0.20 g/100 g, and MCL, 7.76 ± 0.21 g/100 g), followed by 80% (*v/v*) ethanol (YCL, 8.47 ± 0.20 g/100 g, and MCL, 7.25 ± 0.14 g/100 g) and water (YCL, 5.51 ± 0.24 g/100 g, and MCL, 6.16 ± 0.42 g/100 g).

Furthermore, extraction yield of *C. latifolia* mixed-age leaves and *C. latifolia* roots extracted using Soxhlet extraction in 80% (*v/v*) ethanol is shown in Figure 5(b). Our data showed CL extract (14.75 ± 0.20 g/100 g) produced a slightly higher yield compared to CR extract (11.44 ± 0.38 g/100 g), suggesting an insignificant difference between the yields of two extracts. Additionally, Soxhlet extraction demonstrated a better extraction of ethanolic leaves compared to sequential method, with the ethanolic Soxhlet yield (14.75 ± 0.20 g/100 g) being roughly two times more than that of the sequential ethanolic leaf extracts (MCL, 7.25 ± 0.14 g/100 g, and YCL, 8.47 ± 0.20 g/100 g).

### 3.2. Phytochemical Contents by Chemical Assays

#### 3.2.1. Qualitative Phytochemical Screening

Phytochemical screening for sequential extracts of *C. latifolia* young and matured leaves was carried out (results in Table 1(a)), which revealed the presence of terpenoids and glycosides, but no alkaloids in the extracts. Saponins and tannins were also present in all the extracts, except for the hexane and dichloromethane extracts. Similarly, results for ethanolic *C. latifolia* leaves of mixed-age and *C. latifolia* root extracts also showed presence of all the phytochemicals, except for alkaloids (see Table 1(b)).

The qualitative analyses to screen the presence of phenolic and flavonoid were not conducted in this study. However, a quantitative study of total phenolic and flavonoid contents was performed, where the results are shown in Section 3.2.2.

#### 3.2.2. Quantitative Total Phenolic and Flavonoid Contents

**Figure 6:**
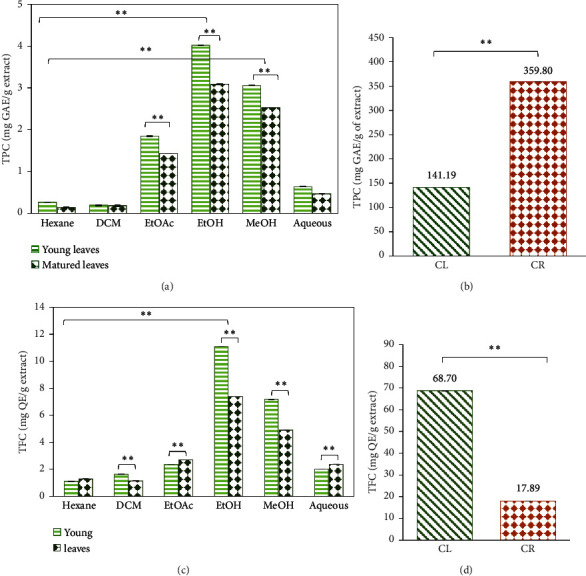
(a) TPC of sequential extracts at 500 mg/L, (b) TPC of ethanolic CL and CR extracts at 1000 mg/L, (c) TFC of sequential extracts at 500 mg/L, and (d) TFC of ethanolic CL and CR extracts at 1000 mg/L. The statistical analysis showed significant differences (^*∗∗*^*p* < 0.01) between the extracts.

The quantification of TPC and TFC in the sequential extracts of YCL and MCL and ethanolic extracts of CL and CR is presented in Figure 6.

A comparison study of TPC and TFC between sequential extracts at 500 mg/L showed high phenolic and flavonoid contents in EtOH-YCL (4.02 ± 0.004 mg GAE/g of extract and 11.083 ± 0.004 mg QE/g extract, respectively). This was followed by EtOH-MCL with TPC of 3.087 ± 0.002 mg GAE/g of extract and TFC of 7.387 ± 0.004 mg QE/g extract (see Figures 6(a) and 6(c)). The results are supported by a statistical analysis which showed a significant difference of phenolic and flavonoid contents (*p* < 0.05) between YCL and MCL.

While young leaves overall showed a greater TPC and TFC, the next part of the study chose to combine the young and matured leaves of *C. latifolia* for the purpose of simplifying the plant collection process. In the TPC analysis for CL and CR extracts at 1000 mg/L, CR revealed a higher value of 359.80 ± 0.01 mg GAE/g of extract compared to CL (141.19 ± 0.01 mg GAE/g of extract).

However, our TFC analysis demonstrated a contrasting result where CR exhibited a lower reading of 17.89 ± 0.00 mg QE/g extract, while CL yielded 68.70 ± 0.00 mg QE/g extract (Figure 6(d)).

### 3.3. Antioxidant Activities

**Figure 7:**
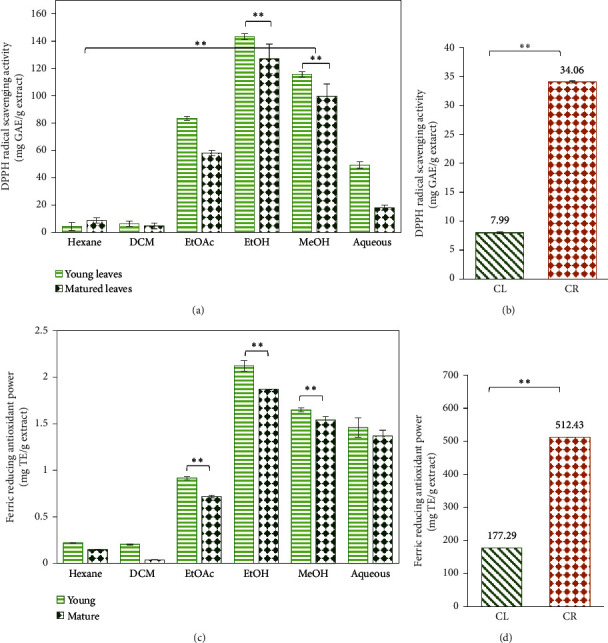
(a) DPPH RSA of sequential extracts of YCL and MCL, (b) DPPH RSA of ethanolic CL and CR extracts, (c) FRAP of sequential extracts of YCL and MCL at 500 mg/L, and (d) FRAP of ethanolic CL and CR extracts at 1000 mg/L. The statistical analysis showed significant differences (^*∗∗*^*p* < 0.01) between the extracts.

The antioxidant activity was evaluated by DPPH radical scavenging activity and ferric reducing antioxidant power assays.  Figure 7 shows the results for the DPPH RSA and FRAP of sequential extracts of young and matured *C. latifolia* leaves and ethanolic extracts of *C. latifolia* leaves and *C. latifolia* roots.

Among the examined sequential extracts, ethanolic extracts showed the highest DPPH radical scavenging activity with YCL having RSA of 143.4 ± 2.1 mg GAE/g of extract and MCL at 127.2 ± 10.8 mg GAE/g of extract (see Figure 7(a)). The antioxidant activity by FRAP assay also found ethanolic extracts to be the most effective in reducing Fe^3+^/ferricyanide complex to ferrous Fe^2+^ by donating free electrons (YCL, 2.120 ± 0.067 mg·TE/g extract, and MCL, 1.868 ± 0.011 mg·TE/g extract) (see Figure 7(c)). Simultaneously, ethyl acetate, methanolic, and aqueous extracts demonstrated promising antioxidant activity in DPPH radical scavenging activity and FRAP assays, suggesting the efficacy of high polar solvents in extracting antioxidants. Our findings on the antioxidant activities of sequential extracts of YCL and MCL show consistency with our TPC and TFC results which could suggest that phenolic and flavonoid compounds are the primary antioxidants. This was supported by a statistical analysis which resulted in a positive correlation of DPPH RSA and FRAP with TPC and TFC (see Table 2).

In the next study, we evaluated the antioxidant activity of CL and CR extracts. Results demonstrated that CR exhibited the most promising antioxidant activity with the highest RSA of 34.06 ± 0.14 mg·GAE/g extract and greatest FRAP activity at 512.43 ± 0.05 mg·TE/g extract. This was followed by CL extract with RSA of 7.99 ± 0.09 mg·GAE/g extract and FRAP of 177.29 ± 0.33 mg·TE/g extract.

Our findings on the antioxidant activities of ethanolic CL and CR extracts showed moderate correlation with TPC (*r* = 0.76), but a negative correlation with TFC (*r* = −0.16) (see Table 2). CR extract with a lower value of TFC (17.89 ± 0.00 mg·QE/g extract) presented a greater antioxidant activity, while CL with the higher TFC (68.70 ± 0.00 mg·QE/g extract) showed a lower RSA and reducing power as shown in Figures 7(b) and 7(d). This indicates that flavonoids have little effect on the antioxidant activity and other major phenolic compounds in the ethanolic extracts may play a major role instead.

### 3.4. Antidiabetic Activity by Chemical Assays

**Figure 8:**
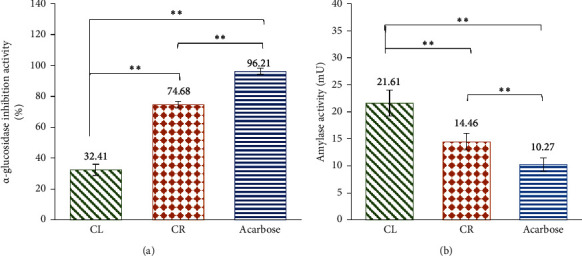
(a)∝-Glucosidase inhibition and (b)∝-amylase enzyme activity of *C. latifolia* leaves (CL) and *C. latifolia* roots (CR) and positive control, acarbose, added as comparison at 200 mg·L^−1^. Results are expressed as % for ∝-glucosidase inhibition activity, while ∝-amylase enzyme activity is expressed in nmol/min/mL (mU). The statistical analysis showed significant differences (^*∗∗*^*p* < 0.01) between the extracts and positive control, acarbose.

Preliminary screening of ethanolic *C. latifolia* leaf and *C. latifolia* root extracts for ∝-glucosidase inhibitory activity and ∝-amylase enzyme activity was carried out at 200 mg·L^−1^, and the results of the study are shown in Figure 8.

Of all the individual extracts, CR exhibited the highest ∝-glucosidase inhibitory activity at 74.68 ± 2.14%, which is comparable with that of a marketed antidiabetic drug, acarbose (96.21 ± 2.03%) (see Figure 8(a)). Meanwhile, CL extract showed a lower ∝-glucosidase inhibition activity with a percentage inhibition of 32.40 ± 1.13%.

Furthermore, the data for ∝-amylase enzyme activity are expressed as nmol/min/mL (milliunit (mU)), where one unit of amylase refers to the amount of amylase that links ethylidene-pNP-G7 to produce 1.0 *μ*mol of p-nitrophenol per unit time. In this study, the lower the ∝-amylase enzyme activity is, the more effective the sample extract is in inhibiting the enzyme. As a result, CR extract was shown to be the most effective extract to inhibit the ∝-amylase activity (14.46 ± 1.54 mU) and is comparable to that of acarbose (10.27 ± 1.21 mU) (see Figure 8(b)). Similarly, as observed in ∝-glucosidase inhibitory activity, CL extract exhibited a lower capacity in delaying the hydrolysis of starch complex compared to CR extract, where it displayed a higher ∝-amylase enzyme activity at 21.61 ± 2.40 mU.

### 3.5. Animal Studies

#### 3.5.1. Antihyperglycemic Activity on Animal Model

In this present study, ethanolic *C. latifolia* mixed-age leaf extract was investigated for its effect on the blood glucose levels of alloxan-induced male Wistar rats, followed by the evaluation of body weight percentage and intraperitoneal glucose tolerance test (IPGTT).

**Figure 9:**
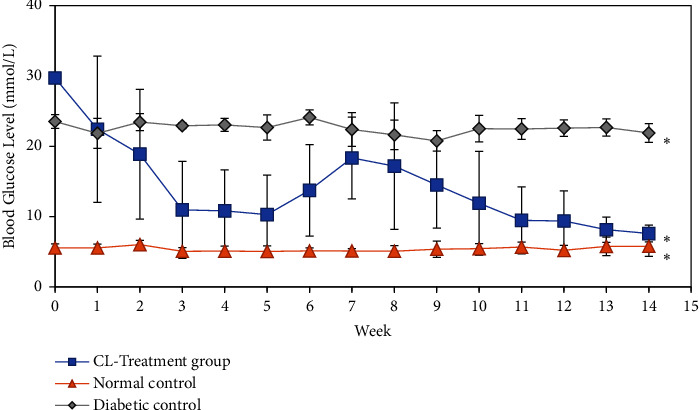
Blood glucose level in mmol/L of normal untreated control, diabetic control, and *Curculigo latifolia* leaf (CL) extract treatment group measured every week for a period of 14 weeks. ^*∗*^*p* < 0.05 indicates significant differences between the experimental groups. Error bars denote standard deviation between BGL readings of animals (*n* = 6).

*(1) Blood Glucose Level and Body Weight Percentage*. Blood glucose readings of animals in normal control, diabetic control, and CL-treated groups are displayed in Figure 9. The normal untreated group showed consistent blood glucose levels between 5.05 ± 0.80 to 6.02 ± 0.59 mmol/L, while alloxan-induced diabetic control group continued to show high blood glucose concentrations between 20.76 ± 1.46 and 23.52 ± 0.99 mmol/L. The results obtained from these control groups are used as reference for normal and diabetic blood glucose levels to compare with our findings on CL-treated group. As a result, BGL of the extract-treated diabetic rats demonstrated a significant decrease (*p* < 0.05) at the first 5 weeks after induction of alloxan and treatment with 250 mg/kg b.w. CL extract, attaining a reading of 7.6 ± 1.21 mmol/L by the final week of treatment.

Subsequently, the body weights of the animals were monitored on a weekly basis until the end of the experimental period. The data are represented as percentage change in body weight as displayed in Table 3. Diabetic animals receiving treatment of 250 mg/kg b.w*. C. latifolia* leaf extract were shown to have a significant body weight gain (*p* < 0.05), reaching a percentage change of 45.83 ± 7.33% at week 14. The results were comparable to that of the normal control group which showed a body weight change of 32.50 ± 3.40% by the end of the experimental period. Meanwhile, the alloxan-induced diabetic group displayed a reduction in body weight of −10.16 ± 5.35%, which is a characteristic sign of diabetes.

**Figure 10:**
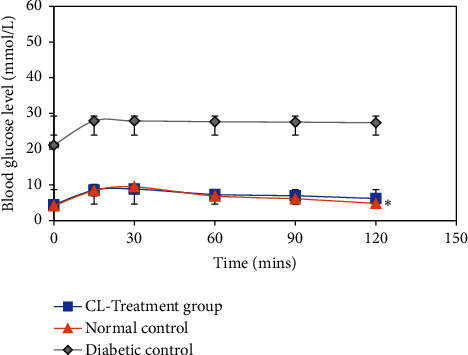
Blood glucose level in mmol/L portraying glucose tolerance of the animals in normal control, diabetic control, and CL treatment groups at 0, 15, 30, 60, 90, and 120 minutes. ^*∗*^*p* < 0.05 indicates significant differences in the blood glucose levels between CL-treated group and alloxan diabetic control group at 120 min. Error bars denote standard deviation between BGL readings of animals (*n* = 6).

*(2) Intraperitoneal Glucose Tolerance Testing (IPGTT)*. IPGTT was also carried out on the animals of normal control, diabetic control, and CL-treated groups at the end of their 14-week experimental period, where their fasting blood glucose levels were measured to evaluate their glucose tolerance. The results are displayed in Figure 10, where changes in blood glucose levels are monitored at a time interval of 15, 30, 60, 90, and 120 minutes. The initial mean BGLs at 0 minutes for fasting animals in normal control and CL treatment groups were 4.12 ± 0.48 and 4.43 ± 0.39 mmol/L, respectively, which are within the range of normal fasting blood glucose concentrations (3.9 to 5.6 mmol/L). Meanwhile, the initial fasting blood glucose level of diabetic control group was 21.12 ± 1.59 mmol/L which exceeded the diabetic threshold value of 7.0 mmol/L. IPGTT of CL treatment group was comparable with that of normal control group, where the final BGLs at 120 minutes were 4.88 ± 0.90 mmol/L and 4.82 ± 0.58 mmol/L, respectively. Moreover, the results were significantly lower than the blood glucose readings of diabetic rats (27.4 ± 0.52 mmol/L at 120 minutes).

#### 3.5.2. Wound-Healing Activity on Animal Model

**Figure 11:**
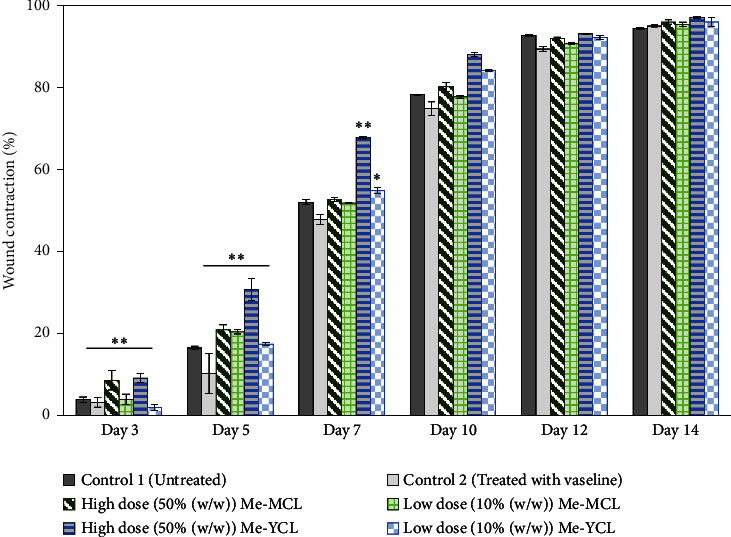
Average wound contraction of control group 1 (untreated), control group 2 (treated with 100% (w/w) Vaseline), high dose matured leaf extract (50% (w/w) Me-MCL), high dose young leaf extract (50% (w/w) Me-YCL), low dose matured leaf extract (10% (w/w) Me-MCL), and low dose young leaf (10% (w/w) Me-YCL) extract on Day 3, 5, 7, 10, 12, and 14. ^*∗*^*p* < 0.05 and ^*∗∗*^*p* < 0.01 indicate significant differences between experimental groups and control group 1 (untreated).

In our preliminary wound-healing study, methanolic extracts of young (Me-YCL) and matured *C. latifolia* (Me-MCL) leaves were investigated for their wound-healing activity on male Wistar rats of 8–10 weeks old. The wound area of treated animals was topically applied with high dose 50% (*w/w*) Me-YCL, high dose 50% (*w/w*) Me-MCL, low dose 10% (*w/w*) Me-YCL, and low dose 10% (*w/w*) Me-MCL. Subsequently, wound contractions of the animals were measured on Day 3, 5, 7, 10, 12, and 14 (see Figure 11). High dose 50% (*w/w*) Me-YCL showed a prominent activity in wound healing when compared to control group 1 (untreated) at the beginning of the study. When comparing the results between untreated control group and the 50% (*w/w*) Me-YCL, a difference in wound contraction on Day 3, 5, and 7 was evident, where untreated group reported the measurements of 3.8 ± 0.7%, 16.5 ± 0.5%, and 52.0 ± 0.6%, respectively, whereas 50% (w/w) Me-YCL reported the measurements of 9.1 ± 1.3%, 30.7 ± 0.5%, and 67.8 ± 0.0%, respectively. Additionally, 50% (w/w) Me-MCL also showed promising wound-healing progression on Day 3, with a wound contraction of 8.6 ± 2.4%, while that of untreated control group was at 3.8 ± 0.7%.

At the end of the experimental study (between Day 12 and Day 14), all the experimental groups had eventually reached more than 90% of wound contraction. However, none of the treated groups showed a prominent difference in wound contractions when compared with the untreated control group.

Table 4 shows the physical observation of the wound-healing progression for all the groups on Day 1, 3, 5, 7, 10, 12, and 14. Scab formation began to occur on Day 3 for all the experimental groups (including control). Meanwhile, detachment of these scabs can be seen on Day 10 for 50% (*w/w*) Me-MCL, 50% (*w/w*) Me-YCL, 10% (*w/w*) Me-MCL, and 10% (*w/w*) Me-YCL, while the scabs in the control groups were retained until Day 12. Additionally, wound size of animal treated with 50% (*w/w*) Me-YCL appeared to be the most diminished on Day 10 compared to the rest of the experimental groups, which shows consistency with its reported wound contraction seen in Figure 11.

## 4. Discussion

### 4.1. Extraction Yield of Crude Extracts

In our sequential extraction of *C. latifolia* young and matured leaves, the use of high polar solvents presented a higher extraction yield compared to low polar solvents, with 80% (*v/v*) methanol being the best, followed by 80% (*v/v*) ethanol and water. Our results show consistency with a previous study by Nur et al. [21] which reported high polar solvent, specifically 70% (*v/v*) ethanol, to be effective in dissolving polar compounds and producing high extraction yield of *C. latifolia* leaf extract. In another finding by Ooi et al. [22], extraction yield of *C. latifolia* rhizome aqueous extract was the highest compared to low polar solvents such as hexane and ethyl acetate.

Solvents with high polarity such as ethanol, methanol, and water are commonly used in the extraction of natural products to extract polar compounds and often result in high percentage yield [23]. Additionally, hydro-alcohols are also common extraction solvents which are efficient in extracting a wide range of compounds including hydrophilic (water-soluble) and lipophilic (fat-soluble) substances [24]. The polarity of ethanol solvent is more consistent in dissolving polar components such as phenolic compounds, glycosides, steroids, alkaloids, polysaccharides, and phospholipids [21, 24]. Meanwhile, water as a solvent system is effective in extracting water-soluble components such as saponins, tannins, terpenoids, and anthocyanins [25].

Different extraction methods can also affect the percentage yield, as our results demonstrated Soxhlet method to give a higher yield of ethanolic extracts compared to sequential extraction. The use of high temperature in the Soxhlet method allows a reduction in water density and viscosity, further increasing the mass transfer of the solvent into the sample matrix [26]. However, increasing the temperature to a certain point can also cause the solution to saturate, preventing the dissolution of solids, and subsequently, reducing the extraction yield [26]. This was evident in the study by Zabidi et al. [26], where the optimum temperature for the extraction of *C. latifolia* roots was at 180°C and as the temperature increased to 200°C, its percentage yield decreased. Based on our study, extraction of *C. latifolia* plant using the Soxhlet method and high polar solvents such as 80% (*v/v*) ethanol, 80% (*v/v*) methanol, and water result in the best extraction yield.

### 4.2. Phytochemical Contents by Chemical Assays

In the present study, phytochemicals such as tannins, saponins, glycosides, and terpenoids were found in all the extracts (refer to Table 1). These secondary metabolites are known for their protective role against diseases and infections such as diabetes, cancer, and cardiovascular diseases. Previous studies have found *C. latifolia* leaves to contain several phytochemicals such as cinnamic acid, berberine, and glycosides which may have played a part in the scavenging of free radicals and antidiabetic activity [27].

Furthermore, our findings showed that using different extraction solvents resulted in different phytochemical profiles, with diverse compositions and concentrations of active compounds. Generally, nonpolar solvents like *n*-hexane and dichloromethane have the tendency to extract nonpolar compounds, whereas polar solvents such as methanol and water would most likely extract polar substances. This accounts for the observed difference in phytochemical response, and as expected, the *C. latifolia* leaves extracted using weakly polar solvents, *n*-hexane and dichloromethane, showed the least extraction of polar-majority phenolic and flavonoid compounds.

Aside from extraction solvents, the maturity of leaves was also found to have an impact on the extraction of phytochemicals. In this study, young leaves of *C. latifolia* were shown to contain a higher content of phenolic and flavonoid compounds compared to matured leaves. This is supported by a previous finding which reported the decrease of flavanols and phenolic acid content upon leaf aging [28]. Nevertheless, it is also demonstrated that some valuable compounds such as rutin were found accumulated in matured leaves which could be beneficial for treating inflammation, lowering cholesterol levels, and improving blood circulation [28]. Therefore, both young and matured can be valuable components of a diverse and nutritious diet which can help in maintaining the overall health and well-being of humans.

In the next study, mixed-age *C. latifolia* leaves and *C. latifolia* roots in 80% (*v/v*) ethanol were analysed. Our results showed that CR had the most promising TPC compared to CL extract. There has been a previous study that revealed CR extract to contain phenolic compounds including scandenin, pomiferin, phloridzin, and mundulone [26]. The presence of these phenolics was described to play a key role in its antioxidant [29] and antidiabetic [30] activities. However, there are still limited studies that have reported on the phenolic content of *C. latifolia* root extract and further extensive investigation is needed to assess the extent of the biological capacities of these chemical compounds.

The discrepancy in TPC results between CR and CL could be due to various reasons, including environmental biotic and abiotic stresses [29]. Additionally, the use of heat in the Soxhlet extraction process may have also resulted in the loss of some important volatile phenolic compounds in CL extract. Therefore, modifications to the study such as employing different extraction methods and varying the ratio for water-ethanolic solvents could be worthwhile to extract other potential bioactive phytochemicals that could exert various biological activities such as antidiabetic, anti-inflammatory, wound-healing, and antimicrobial activities.

Interestingly, our findings revealed TFC of CR to be lower than CL (see Figure 6(d)), indicating that the majority of phytochemical compounds present in CR extract may be composed of phenolics rather than flavonoid compounds. However, flavonoid compounds existing in CR could still impart useful medicinal properties. For instance, Ullah et al. [31] described that flavonoids can serve as exogenous antioxidants, which can form free radicals to less reactive species through mechanisms such as suppressing nitric oxide synthase activity, inhibition of xanthine oxidase activity, modulation of channel pathways, or interacting with other enzyme systems. Quercetin, a potent antioxidant flavonoid, has been widely reported for its versatile protective abilities, including anti-inflammatory, antihypertensive, and antiobesity activities [32]. Additionally, the active compound has shown neuroprotective effects through its ability in suppressing neuroinflammation and promoting memory, learning, and cognitive functions [33]. As for CL extract, its greater TFC is consistent with a previous study by Umar et al. [29], of which it was indicated that flavonoids are generally accumulated in the aerial parts of the plant to provide protection against pathogenic attacks or solar radiation.

The phytochemicals found in CL and CR extracts possess significant potential in supplementing dietary needs that could contribute to the enhancement of overall health and in the treatment of various chronic diseases or health conditions [34]. Further extensive studies on these bioactive compounds are needed to gain more understanding into their properties, potential application, and mechanism of actions. This includes investigating the bioactivity of their isolated active compounds and elucidating the molecular pathways through which these compounds impact the cellular processes.

### 4.3. Antioxidant Activities

Antioxidants play a crucial role in the protection of cells against free radicals that are implicated in the pathogenesis of many diseases such as cancer, inflammation, DNA damage, lipid peroxidation, cardiovascular disease, and diabetes [35]. Many phytochemicals have antioxidant properties that can quench free radicals, chelate catalytic metals, and subsequently terminate chain reactions and inhibit oxidation reactions [35, 36]. Consequently, these antioxidant agents can stimulate the immune system, block the formation of carcinogen, and prevent the production of estrogen implicated in breast cancer [36].

Among the sequential extracts, ethanolic YCL and MCL extracts exhibited the most effective DPPH radical scavenging activity and ferric reducing power, while hexane and dichloromethane extracts were the lowest. Our findings showed consistency with our TPC and TFC results which could suggest that the polar polyphenol compounds are the primary antioxidants that are responsible in scavenging free radicals and reducing ferric ions. Another observation from Figures 7(a) and 7(c) can be seen where YCL demonstrated a greater antioxidant activity compared to MCL. This is supported by the statistical analysis that showed a significant difference of antioxidant activity (*p* < 0.05) between the young and matured leaves. Furthermore, our results agree with a previous finding, which reported that immature leaves overall exhibited higher antioxidant capacities through DPPH, FRAP, 2,2′-azino-bis-(3-ethylbenzothiazoline-6-sulfonic acid) diammonium salt (ABTS), and nitric oxide chemical assays [37]. In addition, the study by Thi and Hwang [38] described that during leaf maturation, different oxidative metabolism and antioxidative strategies in plant tissues take place. Their findings reported younger *Aronia* leaves to have higher antioxidant activity which is consistent with our results on YCL [38].

While ethanolic YCL and MCL extracts showed positive results, our next study demonstrated ethanolic CR extract to exhibit a more prominent radical scavenging activity and ferric reducing antioxidant power. Despite CR's very low TFC, it can be hypothesised that its phenolic compounds were the major contributors in scavenging DPPH radicals and reducing ferric ions. In a previous study by Zabidi et al. [26], the presence of phenolic compound, hydroquinone, in *C. latifolia* root extract was suggested to have contributed to its strong antioxidant activity. The active compound and its derivatives contain hydroxyl groups that can exert a strong radical scavenging activity between 93% and 97%, comparable to a known antioxidant, ∝-tocopherol (95%) [39]. Moreover, cinnamic acid in the extract may have also played a major role in the antioxidant activity, where its hydroxyl groups can donate hydrogen or electron, further neutralising free radicals [40].

Overall, ethanolic extracts of *C. latifolia* leaves and *C. latifolia* roots showed potent antioxidant capacity that is important in preventing degenerative diseases such as cancer, diabetes, inflammation, and obesity. This activity may be related to the presence of active phenolic compounds in the extracts that could absorb and stabilise highly reactive free radicals. However, more studies are still needed to further validate this study. Future recommendations which can potentially result in the extraction of more potent antioxidants may include the use of other polar solvents, extraction methods, and different proportions with aqueous solvent.

### 4.4. Antidiabetic Activity by Chemical Assays

The antidiabetic potential of *C. latifolia* leaves and *C. latifolia* root extracts was investigated via ∝-glucosidase and ∝-amylase inhibitory assays. The extracts were evaluated on their capacity to inhibit the activity of the two enzymes that are involved in the digestion of carbohydrates and starch which usually causes the increase of postprandial (after-meal) hyperglycemia in diabetic patients [41]. Postprandial hyperglycemia is characterised by an elevated blood sugar concentration following a meal, and in T2DM, their impaired insulin response leads to a delayed uptake of glucose by the cells, making it difficult to maintain near normal blood glucose readings [42]. Therefore, inhibiting ∝-glucosidase and *α*-amylase is a strategy to control blood sugar levels by regulating the digestion and absorption of carbohydrates in the digestive system.

The present study demonstrated a promising antidiabetic activity of CL and CR extracts through ∝-glucosidase inhibition and ∝-amylase enzyme activities. Among the individual extracts, CR extract stood out the most by showing marked ∝-glucosidase and ∝-amylase inhibition activities that were similar to that of a marketed antidiabetic drug, acarbose. This indicated that CR have promising active components that can effectively inhibit these two enzymes which could further help in regulating postprandial hyperglycemia and lowering the risk of developing diabetes [43]. The extract's phenolic and flavonoid contents and phytochemical compounds such as saponins, tannins, terpenoids, and glycosides may have individually or synergistically been involved in the mechanism of action [44]. This agrees with the study by Umar et al. [29] which demonstrated a significant correlation between high phenolic content and ∝-glucosidase inhibition activity. The functional group, configuration, substitution, and number of hydroxyl groups of these phytochemical constituents are the key elements in the attachment to the binding sites of ∝-glucosidase and ∝-amylase enzymes [45]. This leads to a more regulated rate at which carbohydrates and starch are broken down and subsequently would result in the improvement of insulin sensitivity and glucose uptake [46, 47].

On the other hand, CL showed less prominent activity in both ∝-glucosidase inhibition and ∝-amylase enzyme assays than CR. Nonetheless, CL's ability to hinder the action of ∝-glucosidase and ∝-amylase enzymes could still have potential in suppressing the breakdown of carbohydrates in the small intestine and delaying the glucose uptake [41]. Its considerable antidiabetic properties could be greatly beneficial in the development of medication for the treatment of diabetes. Moreover, the dosage of extract may play a key factor in inducing much greater ∝-glucosidase inhibition and ∝-amylase enzyme activities. Increasing the concentration of CL extract may possibly lead to an enhanced outcome; therefore, more studies are needed to determine the right dosage.

### 4.5. Animal Studies

In this present study, we evaluated the antihyperglycemic activity of ethanolic *C. latifolia* leaf (mixed-age) extract on alloxan-induced male Wistar rats. *C. latifolia* mixed-age leaves were selected due to its abundant extraction yield which was sufficient to achieve data for this experimental study. Additionally, there are limited studies conducted on the antihyperglycemic effects of ethanolic *C. latifolia* leaf extract; therefore, findings from our study could be considered novel. Based on our findings, *C. latifolia* leaf extract at 250 mg/kg b.w. was able to reduce the blood glucose levels of diabetic rats to normal within 14 weeks. BGL of the extract-treated diabetic rats demonstrated a significant decrease (*p* < 0.05) at the first 5 weeks after induction of alloxan and attained a reading of 7.6 ± 1.21 mmol/L by the final week of treatment. The results obtained were comparable with those of normal control group by the end of the experiment (see Figure 9), which implies that the treatment of 250 mg/kg b.w. *C. latifolia* leaf extract was effective in exerting glucose-lowering effects, indicating its antihyperglycemic potential.

Subsequently, the animal body weights of CL treatment group had significant improvement compared to the animals in alloxan-induced diabetic control. The percentage body weight gain of the treatment group was highly comparable to that of normal control by week 14. Therefore, the extract showed efficacy towards ameliorating the body weight gain of diabetic animals, suggesting its ability to stimulate insulin secretion by inhibiting lipolysis, proteolysis, and glycogenolysis [48]. Moreover, *C. latifolia* leaf extract showed promising protective effects against diabetes-inflicted weight loss, which is consistent with the significant reduction of blood glucose levels. These effects may be attributed to the presence of phytochemicals such as phenolic acids, flavonoids, terpenoids, tannins, and saponins which have been proven to exhibit antihyperglycemic properties. Thus, our phytochemical screening (see Table 1) has shown *C. latifolia* leaf extract to possess these active components which may facilitate in the synergistic interaction on molecular targets, leading to measurable therapeutic effects. Additionally, their ameliorative effects on blood glucose regulation indicated that the extract was effective in regenerating *β*-cells, stimulating insulin secretion, and maintaining normal glucose homeostasis [49, 50]. Simultaneously, the presence of antioxidants in the extract may help in reducing oxidative stress, further preventing major complications of diabetes such as cardiovascular disease, kidney disease, and nerve damage [51].

Furthermore, intraperitoneal glucose tolerance testing (IPGTT) was carried out on all the experimental groups after their 14 weeks of treatment, of which their fasting blood glucose levels were measured to evaluate their glucose tolerance. From the study, CL-treated diabetic rats showed marked improvement as their blood glucose levels reached 4.88 ± 0.90 mmol/L 120 minutes after being administered with 2 g/kg b.w. D-glucose. The results obtained were comparable with those of normal untreated control throughout the 120 minutes IPGTT experiment, indicating that CL-treated diabetic animals may have achieved normal bodily functions after the 14 weeks of treatment. Meanwhile, BGL readings of diabetic animals maintained above the normal threshold, indicating its impairment in insulin secretion and regulating blood sugar levels.

There have been previous findings that reported on the antidiabetic properties of *C. latifolia* plant. A study by Ishak et al. [30] revealed aqueous extract of *C. latifolia* fruit: root at 200 mg/kg b.w. significantly decreased the glucose level of streptozotocin- (STZ-) induced diabetic rats. The results found that the extract was effective in preventing further disruption of *β*-cells and improved insulin and adiponectin secretions that contributed to the decrease of glucose and lipid levels of the animals [30]. A previous study by Ooi et al. [52] has described that the bioactive compound, curculigoside, may possess antidiabetic properties from its high efficacy in glucose transport activity via stimulation of glucose transporter 4 (GLUT4) levels at the plasma membrane in 3T3-L1 adipocytes. Additionally, cinnamic acid in *C. latifolia* was also indicated to contribute to facilitating insulin secretion by activating the voltage-dependent Ca^2+^ channels, inducing an increase in Ca^2+^ and closure of ATP-sensitive K^+^ channels, without causing membrane depolarisation [27, 53]. Furthermore, *C. latifolia* has been revealed to contain sweet proteins known as curculin and neoculin [54]. These proteins were found to be 500 to 9000 times sweeter than sucrose by weight, and thus they have been used as low-calorie sweeteners and taste modifiers [55]. Their nature as sugar substitutes may have potential as antidiabetic agents. However, no studies have been conducted to confirm the notion; therefore, further studies would be worthwhile to find out their antidiabetic capacity.

Our findings could suggest *C. latifolia* leaves as a potential source for the development of food supplements, which could improve insulin sensitivity, glucose metabolism, and overall health and well-being. However, there is still a lack of understanding on the mechanism of action and the role of phytochemical constituents underlying the bioactivity of the leaf extract as there are very limited studies in this area. Extensive studies involving microscopic anatomy and immunohistochemical analysis on the body organs of animals affected by diabetes such as pancreas, liver, and kidney could therefore offer more information and better understanding of its pathology.

Diabetic patients typically have a higher risk of experiencing complications related to wound healing compared to nondiabetic individuals. Consequently, the next study investigated the wound-healing effects of methanolic young and matured *C. latifolia* leaf extracts on male Wistar rats. Based on our results, high dose 50% (*w/w*) Me-YCL resulted in an enhanced wound repair process due to its higher content of phenolic and flavonoid compounds. The wound contractions between untreated control group and the 50% (*w/w*) Me-YCL were significant (*p* < 0.01) on Day 3, 5, and 7, where untreated group resulted in 3.8 ± 0.7%, 16.5 ± 0.5%, and 52.0 ± 0.6%, respectively, whereas 50% (*w/w*) Me-YCL resulted in 9.1 ± 1.3%, 30.7 ± 0.5%, and 67.8 ± 0.0%, respectively. Wound contraction plays a key role during the healing process as it decreases the wound size and promotes re-epithelization by minimising the distance traveled by migrating keratinocytes [56]. The increased rate of wound contraction in the animals treated with 50% (*w/w*) Me-YCL ointment may be attributed to its phenolic compounds which have antibacterial, anti-inflammatory, and antioxidant properties [57]. Additionally, the antioxidative properties of the leaf extract may have also contributed to the reduction of oxidative stress and subsequently led to the improvement of collagen synthesis, migration of myofibroblast, and proliferation of epithelial cells [58, 59]. Furthermore, by Day 12 and Day 14, percentage wound closure of more than 90% was observed in all the experimental groups, indicating the efficacy of Me-YCL and Me-MCL extracts in wound repair.

The physical observation of the wound-healing progression for all the groups on Day 1, 3, 5, 7, 10, 12, and 14 can be seen in Table 4. Scab formation began to occur on Day 3 for all experimental groups, which may indicate the start of hemostasis, where platelet clots are formed to prevent further bleeding and protect from external contamination [60]. In this phase, fibrin matrix is also generated which strengthens the initial seal and provides a framework for tissue restoration. Subsequently, detachment of these scabs can be seen on Day 10 for all treatment groups, while the scabs in the control groups were retained until Day 12. In this stage, angiogenesis is occurring, where formation of new blood vessel capillaries and granulation tissue is produced [61]. Therefore, for the control groups with the scabs retained for a longer time, proliferation of new epithelial cells was delayed, and hence the rate of wound healing was reduced [58].

The presence of bioactive compounds such as tannins, saponins, terpenoids, and glycosides in *C. latifolia* leaf extracts may have played a key role in the healing process. Their overall bioactivities such as antimicrobial, anti-inflammatory, and antioxidant could have provided a significant contribution to the rate of wound repair. For example, due to the antimicrobial and anti-inflammatory activities in tannins and saponins, they can effectively scavenge free radicals and enhance re-epithelialization and matrix synthesis of the wound area, restricting inflammation that can delay the wound-healing process [62, 63]. As for glycosides and terpenoids, it has been reported that these compounds can promote collagen synthesis, along with fibroblast migration at the early proliferation phase of the wound-healing process [64, 65]. This is supported by the study of another species from the same genus known as *Curculigo orchioides* which showed that the wound-healing activity was improved with the facilitation of the phenolic glycoside compounds from the methanolic root extract such as corchioside A, issocrassifoside G, curculigosaponin A–F, sitosterol, linoleic acid, palmitic acid, and orcinol glucoside [63]. These compounds were suggested to have enhanced the blood supply for vasoconstriction, epidermal cell migration, and re-arrangement of collagen fibers, thus stimulating the wound-healing activity [63].

Our preliminary study revealed a potential in wound-healing activity of methanolic young and matured *C. latifolia* leaf extracts on male Wistar rats. However, some modifications to the study, such as increasing the dosage of extracts and exploring various formulations, can provide further improvements to the wound-healing process. Additional research is also needed to examine the impact of other extracts, such as ethanolic and aqueous extracts. Furthermore, an investigation into the different plant parts of *C. latifolia*, for example, the roots and rhizome, are essential for a comprehensive understanding of the plant's potential effects.

## 5. Conclusions

The present study showed promising phytochemical, antioxidant, and antidiabetic activities in *C. latifolia* leaves and *C. latifolia* roots of Brunei Darussalam. Overall, *C. latifolia* roots presented the most prominent antioxidant and antidiabetic activities due to its phenolic content. Furthermore, ethanolic *C. latifolia* leaves have promising antihyperglycemic properties that could be greatly useful in the treatment of type 2 diabetes. Our study also demonstrated potential wound-healing activity by treatment with methanolic young and matured *C. latifolia* leaf extracts. However, a significant difference in comparison with control untreated group was only observed on Day 3, 5, and 7, and thus modifying the concentration levels of extract can potentially enhance the results. All the extracts studied have demonstrated some degree of bioactivity which could be utilised in the development of new drugs or health supplements for the treatment of various diseases. However, more studies such as their molecular pathways, compound isolation, and structure-activity relationship are needed to understand the mechanism of actions between the active components and their bioactivities.

## Figures and Tables

**Table 1:** Phytochemical screening of (a) sequential extracts of young and matured leaves of *C. latifolia* and (b) *C. latifolia* leaf (CL) and *C. latifolia* root (CR) individual extracts.

(a)						
Solvent	Plant material	Alkaloids	Tannins	Saponins	Terpenoids	Glycosides
Hexane	Young leaves	(−)	(−)	(−)	(+)	(+)
	Mature leaves	(−)	(−)	(−)	(+)	(+)

DCM	Young leaves	(−)	(−)	(−)	(+)	(+)
	Mature leaves	(−)	(−)	(−)	(+)	(+)

EtOAc	Young leaves	(−)	(+)	(+)	(+)	(+)
	Mature leaves	(−)	(+)	(+)	(+)	(+)

EtOH	Young leaves	(−)	(+)	(+)	(+)	(+)
	Mature leaves	(−)	(+)	(+)	(+)	(+)

MeOH	Young leaves	(−)	(+)	(+)	(+)	(+)
	Mature leaves	(−)	(+)	(+)	(+)	(+)

Aqueous	Young leaves	(−)	(+)	(+)	(+)	(+)
	Mature leaves	(−)	(+)	(+)	(+)	(+)

(b)						
Sample extracts		Alkaloids	Tannins	Saponins	Terpenoids	Glycosides

*C. latifolia* leaves (CL)		(−)	(+)	(+)	(+)	(+)
*C. latifolia* roots (CR)		(−)	(+)	(+)	(+)	(+)

(+) and (−) denote the presence and absence of compounds, respectively. DCM: dichloromethane; EtOAc: ethyl acetate; EtOH: ethanol; MeOH: methanol.

**Table 2:** The statistical correlation analysis of antioxidant activities with TPC and TFC of YCL and MCL and CL and CR extracts.

	Pearson correlation	*R* value	Implications
YCL and MCL sequential extracts	TPC vs TFC	0.947	TPC and TFC of YCL and MCL extracts are positively correlated, indicating an interdependence of the two phytochemical groups Antioxidant activities of YCL and MCL sequential extracts are attributed to their phenolic and flavonoid compounds
	TPC vs (DPPH) RSA	0.98	
	TFC vs (DPPH) RSA	0.91	
	TPC vs FRAP	0.83	
	TFC vs FRAP	0.82	

Ethanolic CL and CR extracts	TPC vs TFC	0.51	TPC and TFC are not correlated, indicating an inverse relationship between the compounds Antioxidant activities of ethanolic CL and CR extracts are mainly due to their phenolic compounds
	TPC vs (DPPH) RSA	0.76	
	TFC vs (DPPH) RSA	−0.16	
	TPC vs FRAP	0.88	
	TFC vs FRAP	0.06	

*R* value closer to 1 indicates a stronger correlation.

**Table 3:** Percentage change in body weight of normal control and diabetic control and CL treatment group at week 14 of the experimental period.

Experimental group	Percentage change in body weight at week 14 (%)
Normal control	32.50 ± 3.40
Diabetic control	−10.16 ± 5.35
CL treatment group	45.83 ± 7.33

Statistical data marked significant differences (*p* < 0.05) in percentage body weight change between experimental groups. Data are represented as mean ± standard deviation between body weights of animals (*n* = 6).

**Table 4:** Effects of topical application of 80% (v/v) methanolic *C. latifolia* leaf extracts for 14 days.

	Day 1	Day 3	Day 5	Day 7	Day 10	Day 12	Day 14
Control Group 1 (untreated)	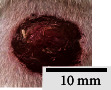	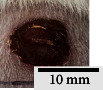	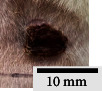	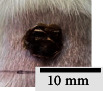	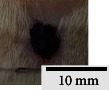	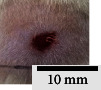	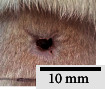

Control Group 2 (treated with Vaseline)	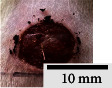	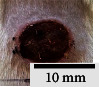	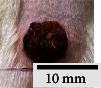	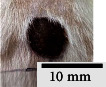	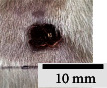	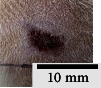	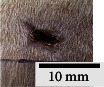

Group 3 (high dose 50% (*w/w*) matured leaf extract)	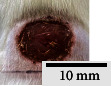	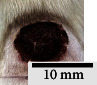	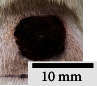	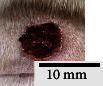	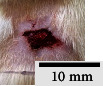	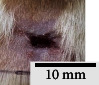	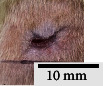

Group 4 (high dose 50% (*w/w*) young leaf extract)	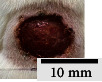	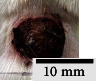	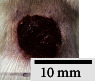	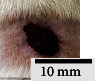	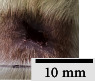	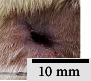	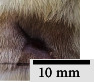

Group 5 (low dose 10% (*w/w*) matured leaf extract)	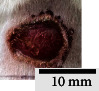	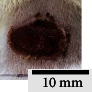	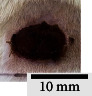	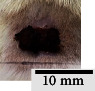	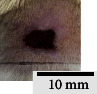	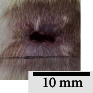	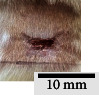

Group 6 (low dose 10% (*w/w*) young leaf extract)	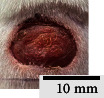	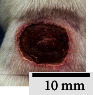	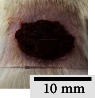	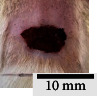	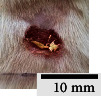	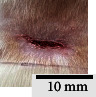	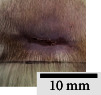

## Data Availability

All data supporting the findings of this study are available within the article. Any additional information can be directed to the corresponding author.
